# Loss of Serum and Glucocorticoid-Regulated Kinase 3 (SGK3) Does Not Affect Proliferation and Survival of Multiple Myeloma Cell Lines

**DOI:** 10.1371/journal.pone.0122689

**Published:** 2015-04-02

**Authors:** Stefan Hausmann, Evelyn Brandt, Carolin Köchel, Hermann Einsele, Ralf C. Bargou, Ruth Seggewiss-Bernhardt, Thorsten Stühmer

**Affiliations:** 1 Department of Internal Medicine II, Division of Hematology and Oncology, University Hospital of Würzburg, Würzburg, Germany; 2 Comprehensive Cancer Center Mainfranken, University Hospital of Würzburg, Würzburg, Germany; Peter MacCallum Cancer Centre, AUSTRALIA

## Abstract

Multiple myeloma (MM) is a generally fatal plasma cell cancer that often shows activation of the phosphoinositide 3-kinase/Akt (PI3K/Akt) pathway. Targeted pharmacologic therapies, however, have not yet progressed beyond the clinical trial stage, and given the complexity of the PI3K/Akt signalling system (e.g. multiple protein isoforms, diverse feedback regulation mechanisms, strong variability between patients) it is mandatory to characterise its ramifications in order to better guide informed decisions about the best therapeutic approaches. Here we explore whether serum and glucocorticoid-regulated kinase 3 (SGK3), a potential downstream effector of PI3K, plays a role in oncogenic signalling in MM cells—either in concert with or independent of Akt. SGK3 was expressed in all MM cell lines and in all primary MM samples tested. Four MM cell lines representing a broad range of intrinsic Akt activation (very strong: MM.1s, moderate: L 363 and JJN-3, absent: AMO-1) were chosen to test the effects of transient SGK3 knockdown alone and in combination with pharmacological inhibition of Akt, PI3K-p110α, or in the context of serum starvation. Although the electroporation protocol led to strong SGK3 depletion for at least 5 days its absence had no substantial effect on the activation status of potential downstream substrates, or on the survival, viability or proliferation of MM cells in all experimental contexts tested. We conclude that it is unlikely that SGK3 plays a significant role for oncogenic signalling in multiple myeloma.

## Introduction

Multiple myeloma (MM) is a haematologic cancer caused by mature, antibody-producing B-cells (plasma cells) [1]. It accounts for ≈ 10% of all haematological malignancies and has an incidence rate in Europe of 4.5-6/100,000/year, affecting primarily the elderly population [2]. Due to ageing societies the incidence is thus continuously rising. Most patients have benefited from the recent introduction of novel therapeutics such as proteasome inhibitors and IMiDs, and survival parameters have shown substantial improvements over the last decade [3,4]. However, it has also become clear that the disease is characterised by a high degree of genetic heterogeneity, potentially due to the long development time from monoclonal gammopathy of undetermined significance (MGUS) to MM [5,6,7]. Truly targeted molecular therapies are thus yet unavailable because actionable and/or broadly relevant therapeutic targets are missing.

One of the growth and survival pathways strongly implicated in MM pathogenesis is the phosphoinositide 3-kinase/Akt (PI3K/Akt) pathway [8,9,10,11,12,13]. In addition to extrinsic activation by microenvironmental factors [14] the pathway is often intrinsically active [10,15]. We have recently shown through isoform-specific knockdown analyses and with isoform-specific pharmacologic inhibitors that the activity of PI3K, and specifically of the isoform p110α, is primarily required to maintain intrinsic Akt activation in MM cell lines [15]. The genetic mechanisms underlying this oncogenic deregulation in MM are not entirely clear as some of the lesions that could potentially be involved, such as *PIK3CA* mutation or *PTEN* deletion, are too rare in this disease to be held fully accountable [16]. Pharmacologic blockade of PI3K-p110α [15] or of Akt [10,11] is toxic to MM cell lines and primary MM cells, with intrinsic Akt activation a good predictor for sensitivity to Akt blockade [10]. In addition, PI3K-p110α or Akt blockade in concert with inhibition of the Ras/MAPK pathway often leads to enhanced MM cell death [11,15]. However, for the Akt-independent MM cell line AMO-1 such a combination effect is seen with PI3K/MEK1,2 inhibition but not with Akt/MEK1,2 inhibition [11,15], arguing for the existence of PI3K-dependent contributions to MM cell survival that can be independent of Akt.

A considerable number of pharmacologic inhibitors for the PI3K/Akt/mTOR axis has recently been developed but translation of preclinical results into useful therapies has remained a challenging task, and—at least for the first two targets—no candidate drug has so far been approved for cancer therapy [17]. However, with the recently reported achievement of clinically relevant responses in some MM patients in a phase I Akt inhibitor trial [18] the possibility for future inclusion of PI3K/Akt inhibition in targeted MM therapies has drawn nearer, and comprehensive knowledge concerning the organisation and ramifications of PI3K-mediated oncogenic signalling in MM is therefore of critical importance for its successful clinical implementation.

The serum and glucocorticoid-regulated kinase 3 (SGK3) belongs like Akt to the AGC group of serine/threonine kinases [19]. In contrast to SGK2, for which very little information is available [19] and to SGK1, which is primarily considered to be regulated in its activity at the genomic level [19,20,21], SGK3 has recently been implicated in some solid cancer cell line models as an Akt-independent transmitter of mutant PI3K-p110α activity [22]. Since SGK3 can potentially complement or substitute for Akt activity downstream of PI3K [19,20,23], such a function would increase the complexity of a signalling network already notorious for its redundancies and regulatory feedback mechanisms [24]. Because the reported pattern of PI3K/Akt activity in multiple myeloma represents a reasonable constellation in which SGK3 signalling could be relevant in at least a subgroup of cases, we decided to analyze its expression in MM and to probe its potential function within the PI3K/Akt signalling system in MM cells.

## Materials and Methods

### Cell lines and primary cells

Human MM cell lines were bought from the German Collection of Microorganisms and Cell Cultures (DSMZ, Braunschweig, Germany) or from LGC Standards (Wesel, Germany). INA-6 cells [25] were a gift from Martin Gramatzki (University Medical Center Schleswig-Holstein, Kiel, Germany). Newly acquired cell lines were immediately expanded to create stock and working banks, which were stored in liquid nitrogen. Subsequently, MM cell cultures were freshly grown from these frozen stocks at 3–4 month intervals. The cell culture parameters were 5% CO_2_ at 37°C in RPMI-1640 medium supplemented with 10% FBS, 1 mM sodium pyruvate, 2 mM glutamine, 100 U/ml penicillin, and 100 μg/ml streptomycin. INA-6 cells were supplied with 2 ng/ml recombinant human interleukin-6. All MM cell line cultures were regularly checked for mycoplasma negativity [26]. The purification of primary MM samples through microbead selection of CD138-positive cells is described in detail in [27]. Patient characteristics and treatment regimens pertaining to the primary MM samples are listed in Table 1.

**Table 1:** Patient characteristics and clinical features.

patient	age	gender	stadium	Ig-type	time since diagnosis (months)	prior therapies
**1**	**65**	**f**	**III B**	**IgG kappa**	**27**	**HD-Melphalan + ABSCT**
**2**	**61**	**m**	**II B**	**IgG kappa**	**22**	**3x HD-Melphalan + ABSCT, RAD, VCD**
**3**	**55**	**m**	**III B**	**IgG kappa**	**2**	**0**
**4**	**67**	**f**	**III A**	**IgA lambda**	**3**	**Dexa, VCD**
**5**	**77**	**m**	**II A**	**IgG kappa**	**82**	**Melphalan-Prednisone, RD, MPV, BPT, VCD, PAD**
**6**	**73**	**f**	**III B**	**IgA kappa**	**0**	**0**
**7**	**75**	**f**	**III A**	**IgG kappa**	**73**	**Dexa, 2x HD-Melphalan + ABSCT, AUY + Bortezomib, BPT, RD, 1x HD-Melphalan + ABSCT, RD**
**8**	**87**	**m**	**III B**	**IgG kappa**	**20**	**Melphalan + Prednisone**
**9**	**59**	**m**	**III B**	**IgG kappa**	**27**	**PAD, 1x HD-Melphalan + ABSCT, Revlimid**

ABSCT: autologous stem cell transplantation

AUY: NVP-AUY922

BPT: bortezomib, prednisone and thalidomide

Dexa: dexamethasone

HD-Melphalan: high dose melphalan

MPV: melphalan, prednisone and bortezomib

PAD: prednisone, adriamycin and dexamethasone

RAD: revlimid, adriamycin and dexamethasone

RD: revlimid and dexamethasone

VCD: bortezomib, cyclophosphamide and dexamethasone

### Ethics statement

Primary MM cells were isolated from bone marrow aspirates of MM patients that were acquired on occasions of routine diagnostic interventions after informed consent of patients (Ethics Committee of the Medical Faculty of Würzburg University, reference numbers: 73/05, 76/13).

### Reagents

Annexin V was prepared following a protocol detailed in [28] and coupled to either fluoresceinisothiocyanate (Sigma, Deisenhofen, Germany; F7250) or to PromoFluor 647 using its N-hydroxysuccinimidyl ester (PromoCell, Heidelberg, Germany; PK-PF647-1). The stealth siRNA against SGK3 (5'-UGCCGAGAUGUUGCUGAAAUGUAUG; representative of bases 1087 to 1111 of the coding sequence for human *SGK3*) was ordered from Life Technologies (Darmstadt, Germany). The sequence is an extended version of a target sequence that was published for a short-hairpin RNA expression construct [22]. The sequence of the (only moderately effective) stealth siRNA against EGFP used in control electroporations was 5'-CGAAGGCUACGUCCAGGGCGCACC. The following small molecule inhibitors were used in this study: PI3K-p110α inhibitor BYL-719 (Active Biochemicals, Bonn, Germany (A-1214)), pan-Akt inhibitor MK-2206 (Active Biochemicals (A-1026)), Akt1&2 inhibitor Akti1,2 (Calbiochem, Schwalbach, Germany (124018)), MEK1&2 inhibitor PD0325902 (Selleck Chemicals, München, Germany (S1036)).

### Electroporation of MM cells and drug treatment of electroporated cells

The protocol for MM cell electroporation with siRNA oligonucleotides and subsequent purification of viable cells is described in detail in [29]. The SGK3 stealth siRNA was used at a concentration of 3 μM in the electroporation mixtures. A stealth siRNA targeting enhanced green fluorescent protein (EGFP) was used in the control electroporations. For drug treatments of electroporated MM cells, the cells were seeded on day 2 post-electroporation into 96 well plates in 100 μl of full medium and at densities of between 15,000 and 50,000 cells per well, dependent on the specific cell line and the intended assay (higher cell numbers for alamarBlue assays, lower numbers for annexin V FACS measurements). Drugs were dissolved in DMSO and kept as frozen stock solutions, from which they were freshly diluted in full medium to either 2x (single drug additions) or 4x (two-drug combinations) working solutions. Of these either 100 μl or 50 μl were added per well of MM cells to yield final volumes of 200 μl. Cells were then cultured for an additional 3–5 days prior to cell death/viability analyses.

### Western blotting and antibodies

Frozen cell pellets were dissolved in Laemmlie-buffer (60 mM Tris-HCl, 10% glycerol, 2% SDS, 10% β-mercaptoethanol, 0.01% bromophenol blue; pH 6.8) (10 μl buffer per 100,000 cells) and subjected to sonication (3–5 s on ice with a UP50H sonicator equipped with an MS1 sonotrode) (Hielscher, Teltow, Germany). Samples were then heated to 89°C for 3 min, spun for 5 min at room temperature and the supernatants used for standard SDS-PAGE with 12% gels. Wet blotting was carried out in Mini Trans-Blot modules (Bio-Rad Laboratories) using nitrocellulose membranes and blotting buffer (20% v/v methanol, 25 mM Tris-HCl, 192 mM glycine, pH 8.6). The following antibodies were used for target detection: anti-β-actin (Sigma-Aldrich, Deisenhofen, Germany; A5316), anti-pan-Akt (Cell Signaling Technology (CST), Frankfurt am Main, Germany; no. 9272), anti-phospho-Akt (Thr320) (CST; no. 2965), anti-phospho-Akt (Ser473) (CST; no. 4058), anti-phospho-ERK1/2 (CST; no. 9101), anti-FOXO1/3A (CST; no. 9464), anti-phospho-GSK-3β (CST; no. 9336), anti-phospho-PDK1 (Ser241) (CST; no. 3438), anti-phospho-PRAS40 (Perbio Science, Bonn, Germany; PA5-17175), anti-phospho-PRAS40 (CST; no. 2997), anti-SGK3 (Santa Cruz Biotechnology, Heidelberg, Germany; sc-166847). Secondary antibody F(ab')2 fragments coupled to horseradish peroxidase and specific for rabbit-IgG (no. 111-036-045) or mouse-IgG (no. 115-036-072) were obtained from Jackson ImmunoResearch, Newmarket, UK.

Phospho-SGK3 antibodies tested but without specific signals in MM cells included products from CST (no. 5642), Santa Cruz Biotechnology (sc-33044), and a custom-made antibody generously provided by Mark Rider (Brussels, Belgium).

A freshly made solution of luminol (2.5 mM), p-coumaric acid (0.2 mM) and H_2_O_2_ (0.01%) in 100 mM Tris-HCl (pH 8.8) was used as reagent for chemiluminescent detection [30].

### Flow cytometry

Cells were washed with PBS, pelleted and resuspended in 200 μl of cold annexin V binding buffer (10 mM HEPES/NaOH, 140 mM NaCl, 2.5 mM CaCl_2_; pH7.4) containing 1 μl of annexin V-PromoFluor 647 solution (see Reagents) and 1 μg/ml propidium iodide. Flow cytometry was performed with a FACSCalibur (BD Biosciences, Heidelberg, Germany). Datafiles were analysed with FlowJo version 8.8.7 (Tree Star, Inc., Ashland, U.S.A.).

### Mitochondrial activity/viability assay

The alamarBlue colorimetric assay was used to determine knockdown and drug effects on the viability (i.e. the collective and interconnected effects on metabolism, proliferation and cell death) of electroporated MM cells. Between 25,000 (AMO-1, JJN-3, L-363) and 50,000 (MM.1s) cells were seeded per well (96-well plates) and each test condition was prepared in triplicate. Measurements and data analyses were performed as described in the manufacturer's manual (MorphoSys, Oxford, UK). The size of effects was calculated relative to DMSO-treated controls (= 100% values).

### BrdU proliferation assay

MM cells electroporated with siRNAs against EGFP (control) or SGK3 were kept in culture for 4 days and were then treated with 5-bromo-2'-desoxyuridine (BrdU) [20 μM] for 2 h. After 2 washes with PBS cells were resuspended in 70% cold EtOH and stored at -20°C until further use. For BrdU immunostaining the cell suspension was thawed, twice washed in cold PBS and incubated for 30 min at room temperature in acid denaturing solution (2N HCl, 0.5% Triton X-100). Cells were collected and resuspended in neutralization solution (100 μM Na_2_[B_4_O_5_(OH)_4_]) (pH 8.5) for 2 min, washed with PBS and resuspended in 100 μl of 0.5% Tween 20 and 1% BSA in PBS (TBP) containing 1 μl of anti-BrdU-Alexa Fluor 647 conjugated antibody (Invitrogen, Carlsbad U.S.A.; A21305). Incubation was at RT in the dark for 2 h. Cells were washed in 0.5% Tween 20, 1% BSA in PBS, resuspended in PBS/propidium iodide (50 μg/ml) and submitted to FACS analysis.

### Data analysis

Dose-effect curves were calculated from at least three independent experiments by non-linear regression analysis (sigmoidal shape, variable slope setting) using GraphPad Prism 3.0 (GraphPad Software, La Jolla, CA, U.S.A.). Regression analysis was not performed for data sets when it was obvious that a dose-effect relationship did not exist (MK-2206 treatment of AMO-1 cells).

## Results and Discussion

### Expression of SGK3 in multiple myeloma cells

**Fig 1:**
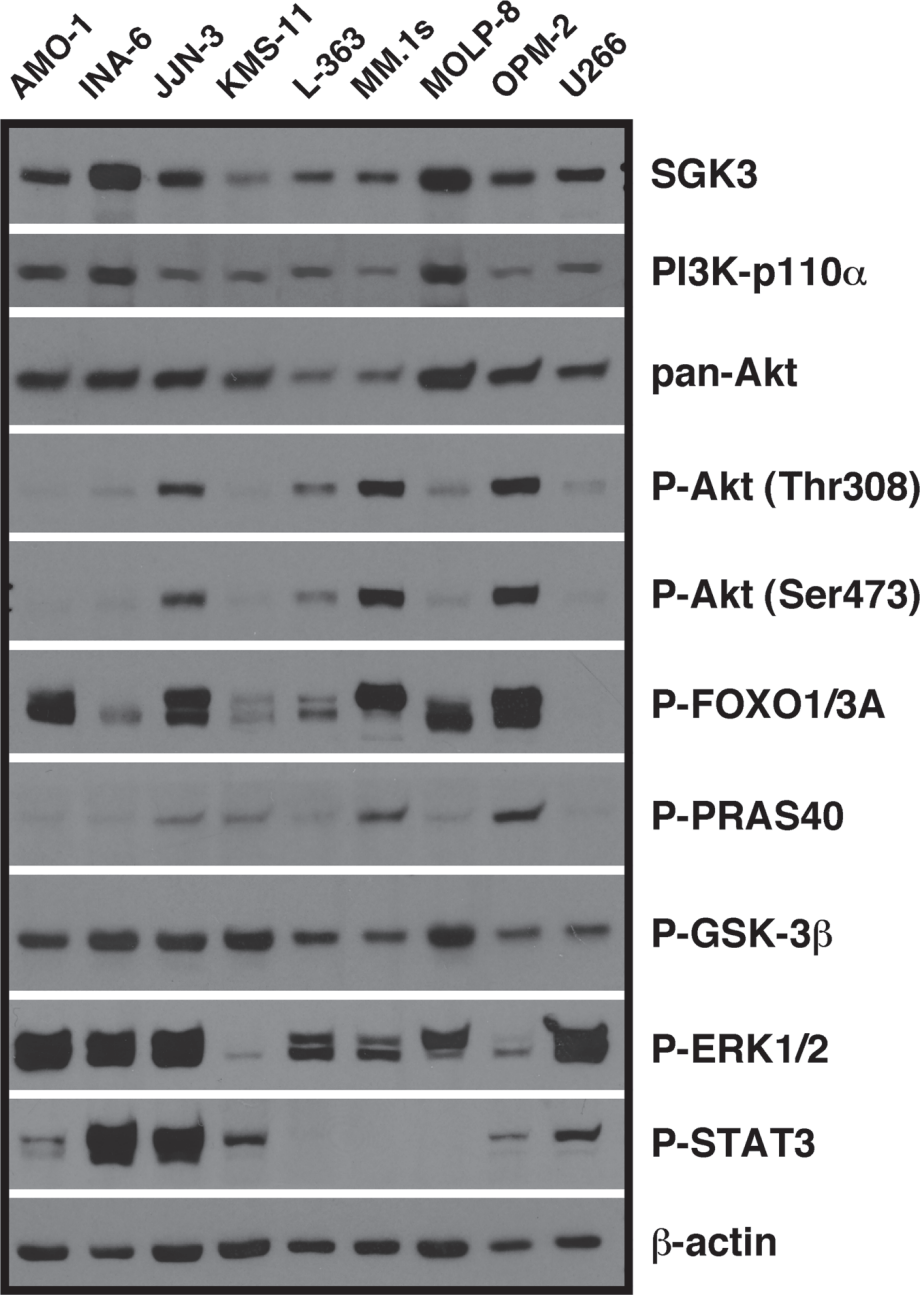
SGK3 expression in relation to (activated) signalling components of the PI3K/Akt system in MM cell lines. Shown are Western blots for PI3K pathway-associated signalling proteins or for their phosphorylated forms. Cells from the MM cell lines indicated were harvested from standard cell culture, the signals are thus representative of steady-state levels in culture. One cell lysate per line was used to load multiple gels. The representative β-actin control derives from the same blot on which SGK3 and P-FOXO1/3A were also stained. Note: the strong phospho-STAT3 signal in INA-6 cells results from permanent supplementation of the culture with recombinant human IL-6.

In order to characterise SGK3 protein expression in MM cells in relation to expression or activation of other components of the PI3K/Akt system we initially performed Western analyses with extracts from MM cell lines and primary MM samples. The level of SGK3 varied considerably between different MM cell lines, but the protein was always detectable (Fig 1). Its expression pattern was similar to that of PI3K-p110α, a major determinant of PI3K to Akt signaling in MM [15,31], and to that of pan-Akt (Fig 1). A major drawback for functional SGK3 analyses is the apparent lack of antibodies for reliable Western assessment of SGK3 phosphorylation under regular culture conditions (i.e. steady-state levels). Although SGK3 phosphorylation across a range of solid cancer cell lines has been reported [22], with the exception of a single cell line [32] such signals have otherwise only been shown in the context of genetically engineered strong protein overexpression [32,33]. We used pairs of SGK3 depleted/control transfected MM samples, in conditions with or without IL-6/IGF-1-mediated stimulation of the PI3K system, to test a number of anti-phospho-SGK3 antibodies in Western blotting. However, we never detected signals that could specifically be attributed to phospho-SGK3 (data not shown, the antibodies tested are listed in the Methods section). Staining for phosphorylated substrates downstream of PI3K (phospho-Akt) and downstream of Akt and, potentially, SGK3 (phospho-FOXO1/3A, phospho-PRAS40, phospho-GSK-3β) confirmed the pattern for phospho-Akt previously reported for MM cell lines [10], with either strong (MM.1s, OPM-2), intermediate (JJN-3, KMS-11, L-363, MOLP-8) or low/undetectable levels (AMO-1, INA-6, U266). Whereas the phospho-PRAS40 pattern closely matched that of phospho-Akt, the patterns of phospho-GSK-3β and phospho-FOXO1/3A showed no such correlation, leaving an Akt independent contribution via SGK3 a possibility (Fig 1). Activity of the Ras/MAPK pathway (represented by phospho-ERK1/2) and the JAK/STAT pathway (represented by phospho-STAT3) was not correlated to activation of any of the PI3K/Akt system components.

**Fig 2:**
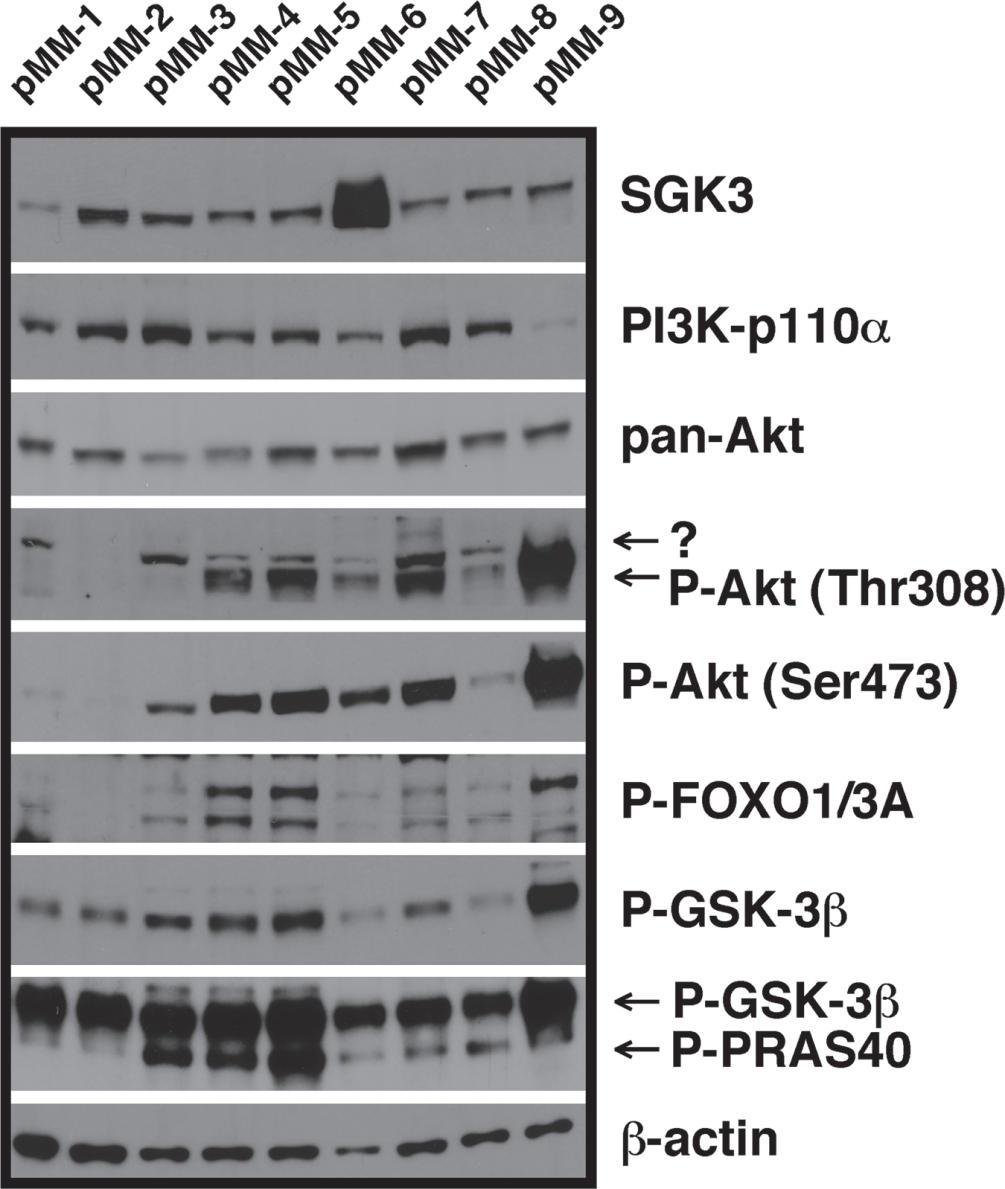
SGK3 expression in relation to (activated) signalling components of the PI3K/Akt system in primary MM cells. Shown are Western blots prepared from frozen pellets of primary MM cells purified by CD138 microbead selection. The material was used to load two gels, with the representative β-actin control belonging to the same blot on which PI3K-p110α, P-FOXO1/3A, P-Akt (Thr308) and pan-Akt were also stained. Of note, the phospho-Akt (Thr308) antibody also stained a slightly larger band in most primary MM samples (marked by "?") that was not visible in any MM cell line. This band runs slightly higher, though, than the SGK3 band as stained on the parallel blot. Staining for P-PRAS40 (CST; no. 2997) was performed after staining for P-GSK-3β. Since both antibodies were raised in rabbit the latter signal reappeared in the P-PRAS40 blot.

SGK3 was also found to be present in all primary MM samples tested (note: the apparently very strong signal in sample pMM-6 is in part the result of rearward smearing off of a strong band that runs slightly lower than full-length SGK3) (Fig 2). Although the phosphorylation levels of PI3K system components showed strong differences between the individual primary samples, the presence of phospho-Akt was more stringently correlated with phosphorylation of downstream substrates than in MM cell lines (Fig 2). Taken together, these analyses showed that SGK3 expression in MM cells appears to be ubiquitous, but that its presence is not obviously correlated to a particular activity pattern of potential downstream substrates.

### Analysis of SGK3 knockdown in MM cells

**Fig 3:**
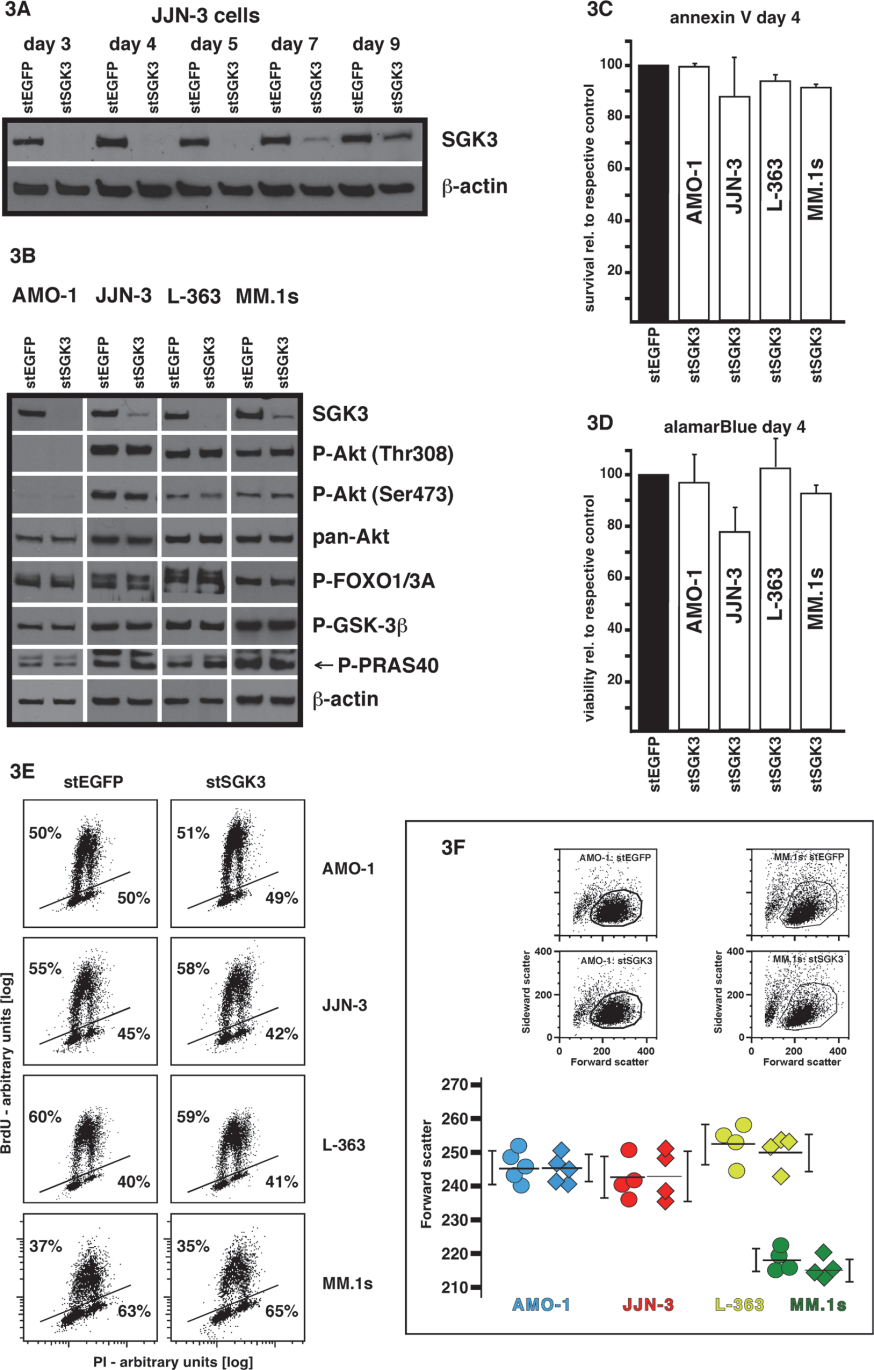
SGK3 knockdown in MM cell lines. A) Western blot showing time-dependence of stealth siRNA-mediated SGK3 (stSGK3) depletion in electroporated JJN-3 cells. A stealth siRNA against enhanced green fluorescent protein (stEGFP) was used in control electroporations. B) SGK3 knockdown and phosphorylation levels of Akt and of potential SGK3 downstream substrates in stEGFP vs. stSGK3 transfected MM cell lines. Cells were harvested at day 3 post-electroporation. For each cell line two gels were run and the representative β-actin control derives from the gel that was also stained for P-Akt (Thr308) (all cell lines) and pan-Akt (L-363, MM.1s) or P-FOXO1/3A and P-GSK-3β (JJN-3). C) SGK3 knockdown does not substantially affect the survival of MM cells. Shown are the results of annexin V/PI staining followed by FACS analysis at day 4 post-electroporation. Relative survival rates are shown, i.e. survival (the annexin V/PI negative fraction) in stEGFP transfected control samples was always set as 100% and survival of the respective SGK3 knockdown samples is shown relative to their cognate controls. At least three independent experiments were performed for each cell line. Error bars denote s.e.m. D) Same experiments as described for C) but viability determined by alamarBlue colorimetric assay. E) Proliferation analysis (one of 2 similar experiments shown) performed on day 4 post-electroporation in MM cell lines treated with the stealth siRNA against EGFP versus stealth siRNA against SGK3. The percentages given above the lines denote the respective share of cells that have incorporated bromo-desoxyuridine (BrdU) during a 2 h BrdU pulse, indicating active DNA synthesis. F) Forward-scatter analysis of stEGFP versus stSGK3 treated cells at day 4 post-electroporation as a measure of cell volume. Top: examples for selection of the live cell fractions of stEGFP and stSGK3 transfected cells based on their forward versus sideward scatter location. Bottom: Distribution of the mean forward scatter values for between 4 and 5 independent experiments (i.e. different electroporations) per cell line. Horizontal black lines mark the mean value of the datapoints shown and vertical black lines indicate the s.e.m.

Because specific pharmacologic inhibitors for SGK3 are not available, we decided to use siRNA-mediated knockdown to investigate the consequences of SGK3 depletion in MM cells. Transient transfection of MM cell lines with RNAi oligonucleotides can efficiently be achieved through electroporation and results in substantial target depletion for at least 5 days [29]. The MM cell lines chosen in our experiments represent an intrinsically phospho-Akt negative line (AMO-1), two that are moderately phospho-Akt positive (JJN-3, L-363) and one that is strongly phospho-Akt positive (MM.1s), and whose phospho-Akt signal is dependent on PI3K-p110α activity [15]. L-363 cells also represent the rare case of an MM cell line with an activating mutation in *PIK3CA*. Electroporation of the stealth siRNA against SGK3 (stSGK3) into MM cells very effectively decreased SGK3 protein levels, and the knockdown remained strong even until day 7 post-electroporation (Fig 3A and 3B; a stealth siRNA targeting EGFP (stEGFP) was used in the respective control transfections). However, Western analyses showed that SGK3 depletion was without effect on the intrinsic activity levels of PI3K/Akt pathway components in all MM cell lines tested (Fig 3B). Furthermore, measurements of cell death (annexin V/PI staining (Fig 3C)) and of mitochondrial activity (alamarBlue assay (Fig 3D)) at day 4 post-electroporation did not reveal differences between SGK3 knockdown cells and their respective controls, except perhaps a slight impairment in the viability of SGK3-depleted JJN-3 cells. Cell cycle/proliferation analyses based on BrdU incorporation into newly synthesized DNA led to identical results for control and SGK3 knockdown cells (assay performed on day 4 post-electroporation, i.e. when SGK3 levels had reached their low point) (Fig 3E). Lastly, the evaluation of FACS-based mean forward scatter readings, again taken for cell samples at day 4 post-electroporation, showed that absence of SGK3 did not affect cell volume. These results are in contrast to, for example, the at least modest effects reported for SGK3 depletion in certain cell lines derived from solid cancers (breast [34], hepatic [35], prostate [36]). On the other hand, siRNA-mediated knockdown of Akt [10] or of PI3K-p110α [15] can substantially affect cell survival/viability in PI3K-p110α/Akt dependent multiple myeloma cell lines like MM.1s, L-363 and JJN-3. The consistent lack of effects in this context therefore argues against a major role of SGK3 on its own for maintenance of MM cell proliferation and survival.

### Analysis of SGK3 knockdown in combination with inhibition of Akt

**Fig 4:**
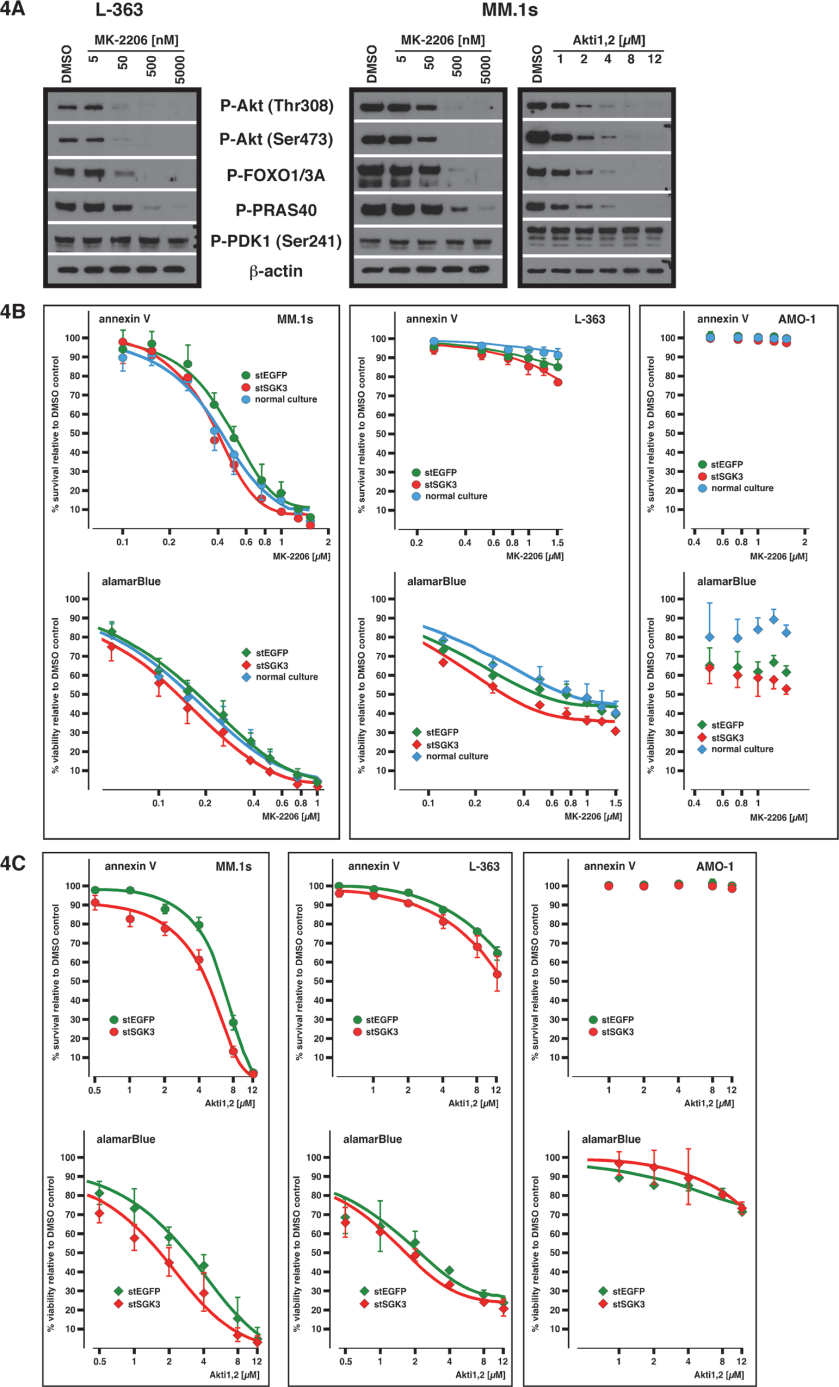
SGK3 knockdown in combination with Akt inhibition. A) Titration of allosteric Akt inhibitors MK-2206 and Akti1,2. MM cells were incubated with the drugs for 30 min prior to harvest for Western blotting. B) Dose-effect curves representing cell death (annexin V) or viability (alamarBlue) assays for MM.1s cells (left), L-363 cells (middle) and AMO-1 cells (right) from either untreated cultures (blue dots), cells electroporated with stealth siRNA against EGFP (green dots) or cells electroporated with stealth siRNA against SGK3 (red dots), and treated with various concentrations of MK-2206 for 3 days. Drugs were added to electroporated cells at day 2 post-electroporation. Cells from untreated cultures were kept at similar densities as those of electroporated cells prior to drug addition. Each dose/effect curve is based on between 3 and 4 independent experiments. Error bars indicate s.e.m. C) Same experimental setup as described in B) but with Akt inhibitor Akti1,2. Each dose/effect curve is based on between 2 and 3 independent experiments. Error bars indicate s.e.m.

We next tested if SGK3 knockdown might be more harmful within the context of simultaneous inhibition of Akt, because both enzymes occupy equivalent positions in signalling pathways downstream of PI3K [19]. SGK3 could thus represent a second pillar of PI3K-mediated growth and survival signalling and its function in MM cells might only be indispensible in conditions of Akt inhibition, or its inhibition might enhance the effects of Akt blockade. We tested two allosteric inhibitors of Akt, MK-2206 [37,38] and the structurally unrelated compound Akti1,2 (also known as Akt inhibitor VIII) [39], both of which bind in a pocket between the pleckstrin-homology domain and the kinase domain and thus prevent Akt phosphorylation/activation [40]. Both compounds abrogated the constitutive phosphorylations of Akt and of the Akt downstream substrates FOXO1/3A and PRAS40 in MM cells, although MK-2206 was effective at lower concentrations than Akti1,2 (Fig 4A; downregulation of phospho-Akt signals with Akti1,2 only shown for the strongly phospho-Akt positive MM.1s cells). These inhibitors did not affect phosphorylation of the upstream kinase PDK-1 at position Ser241, which is presumed to be essential for PDK-1 activity [41]. Both Akt inhibitors were titrated to MM cells electroporated with either stealth siRNA against EGFP (electroporation control cells, green dots in Fig 4B and 4C) or against SGK3 (red dots in Fig 4B and 4C). The drugs were added 2 days after electroporation, i.e. at a time when cell integrity was expected to be fully re-established and when SGK3 knockdown was mostly achieved. Cells were then cultured for another 3 days prior to cell death (annexin V/PI) or viability (alamarBlue) measurements. Additionally, for the MK-2206 treatments, another control titration with cells taken from the regular cell culture (blue dots in Fig 4B) was simultaneously performed to account for potential effects caused by the electroporation procedure. Cell line MM.1s, which is strongly dependent on Akt signalling for survival [10], showed complete killing curves for both inhibitors, whereas the phospho-Akt negative MM cell line AMO-1 was unaffected (lower and not dose-dependent viability values in the AMO-1 alamarBlue assay for treatment with MK-2206 were mostly a consequence of the electroporation treatment per se) (Fig 4B). L-363 cells displayed little to moderate Akt inhibitor-induced acute cell death but were more profoundly affected in their viability, a result in keeping with the role that Akt signalling has for maintenance of metabolic activity [42]. In general, treatment of SGK3 knockdown cells with Akt inhibitors induced marginally higher rates of apoptosis and/or decreases in proliferation/viability compared to respective control cells, but the differences were at best slight and, for MM.1s cells treated with MK-2206, indistinguishable from the effects on untransfected control cells. Taken together, these experiments therefore do not support the notion that SGK3 might act as a redundant safeguard mechanism to maintain growth and survival of MM cells under conditions of Akt blockade.

### SGK3 knockdown in combination with MEK blockade

**Fig 5:**
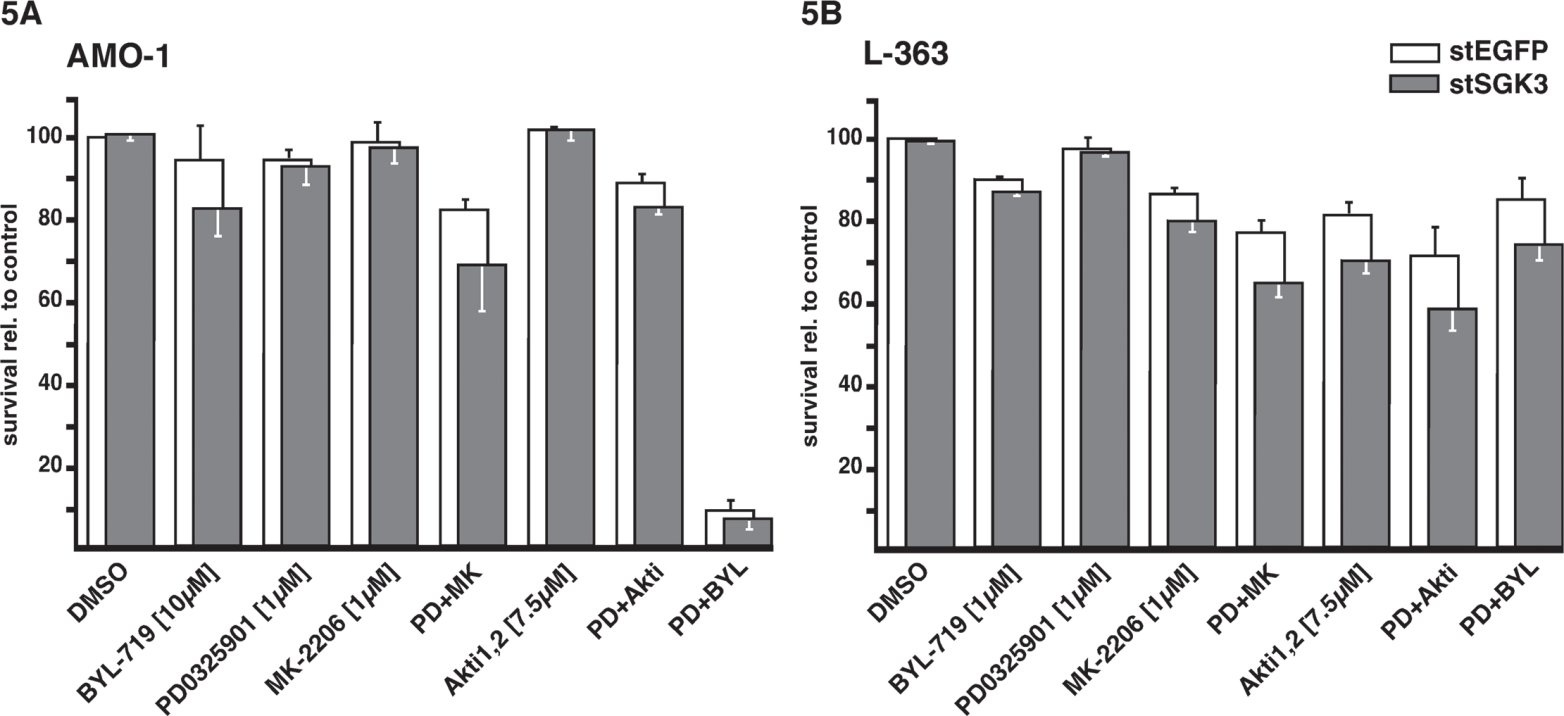
SGK3 knockdown in the broader context of MEK1,2/PI3K blockade. MM cells were electroporated with stealth siRNAs against EGFP or SGK3 and drugs were added at day 2 post-electroporation for a further 3-day incubation. Cell death was measured by annexin V/PI staining and FACS analysis. Error bars denote s.e.m. based on 3 independent experiments. The survival rates were calculated relative to DMSO-treated cells. The absolute survival rates for the experimental pairs in these experiments (DMSO treated stEGFP vs. DMSO treated stSGK3 transfected cells) were 92.7% vs. 93.6% (AMO-1) and 92.7% vs. 91.9% (L-363), i.e. there was no substantial difference between control cells and SGK3 knockdown cells. Titration of BYL-719 and choice of its concentration are detailed in [15]. Of note, the strong synergistic effect observed in AMO-1 cells for the combination of PI3K-p110α inhibitor BYL-719 and MEK1,2 inhibitor PD0325901 is not observed when the PI3K-p110α inhibitor is substituted with an Akt inhibitor (MK-2206 or Akti1,2), but it is also not mirrored by the combination of MEK1,2 inhibition and SGK3 depletion.

We have previously shown that the combined blockade of PI3K and MEK1,2 in the phospho-Akt negative MM cell line AMO-1 leads to strong synergistic induction of cell death. This effect was observed for the combination of pan PI3K/mTOR inhibitor PI103 with MEK1,2 inhibitor PD184352 [11], as well as for the combination of PI3K-p110α inhibitor BYL-719 with MEK1,2 inhibitor PD0325901 [15]. However, no such effect was found for the combination of Akt inhibitor Akti1,2 with MEK1,2 inhibitor PD98059 [10]. We therefore tested if the PI3K-dependent survival signal in this combination blockade with MEK inhibitors is transmitted by SGK3 (Fig 5). AMO-1 cells electroporated with either stealth siRNA against SGK3 (gray bars) or against EGFP (white bars) were kept in cell culture for two days to recover from the effects of electroporation and to downregulate SGK3. Cells were then subjected to single or combination drug treatments for 3 additional days, and apoptosis was measured by annexin V/PI staining and FACS analysis. As expected, the combination of PI3K-p110α and MEK1,2 inhibition (BYL-719 + PD0325901) strongly affected AMO-1 cell survival, whereas each drug alone had little impact, and this effect was not mirrored by combinations of Akt and MEK inhibitor (MK-2206 + PD0325901 or Akti1,2 + PD0325901) (Fig 5A). However, apoptosis induction in PD0325901-treated AMO-1 cells depleted for SGK3 was no more effective than in control cells (Fig 5A, third columns from the left), arguing against SGK3 as the crucial mediator of PI3K-p110α dependence in this cell line. Although SGK3 knockdown cells exhibited slightly increased rates of cell death in some settings (i.e. for treatment with BYL-719 or with PD0325901 + MK-2206; Fig 5A) the effects were always small and within the limits of experimental variation. Taken together, these experiments provided no evidence for a functional role of SGK3 downstream of PI3K-p110α in AMO-1 cells. L-363 cells, which harbour an activating *PIK3CA* mutation, also showed no substantial increases in cell death when pharmacological blockade of MEK1,2, Akt or PI3K-p110α was performed in cells devoid of SGK3 (Fig 5B).

### Serum dependence of MM cells in the context of SGK3 depletion

**Fig 6:**
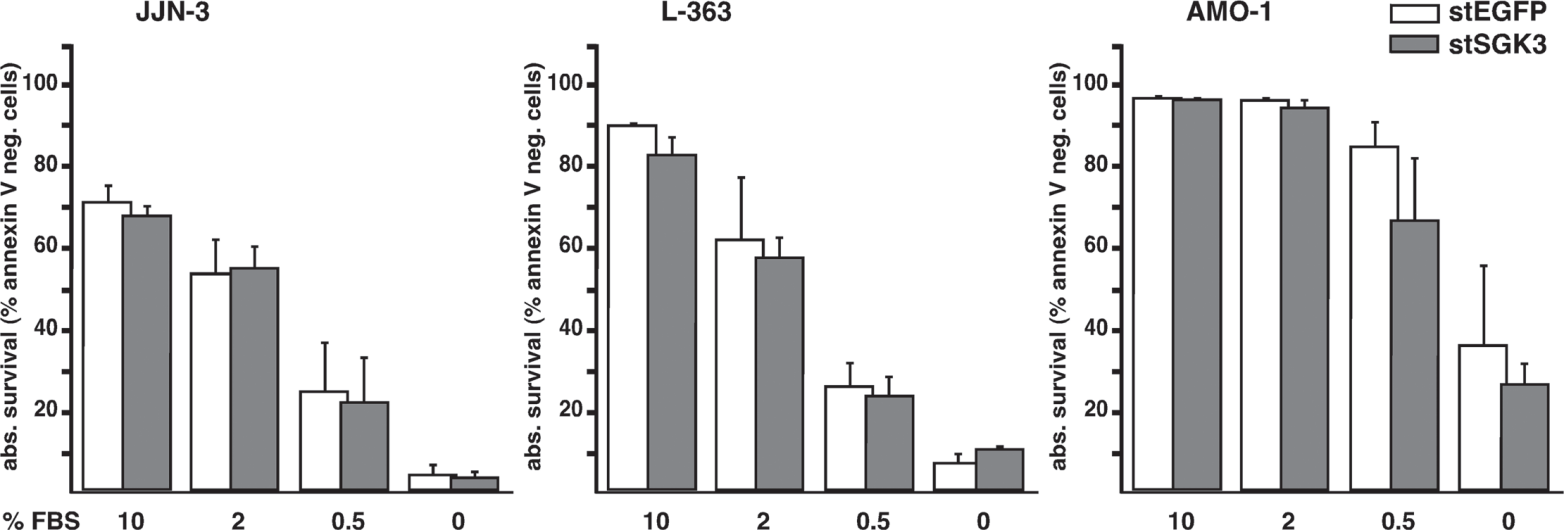
Serum dependence of SGK3 depleted MM cells. MM cells transfected with either stealth siRNA against EGFP (white columns) or against SGK3 (grey columns) were washed three times with PBS at day 2 post-electroporation and resuspended in fresh medium, subsequently adjusted to contain the indicated concentrations of FBS. After further culture for 3 days cell death was determined by annexin V/PI staining and FACS analysis. Error bars indicate s.e.m. based on 4 independent experiments.

We finally tested if SGK3 knockdown had any influence on the survival of MM cells in conditions of serum deprivation, because extrinsic serum-dependent growth and survival signals should at least in parts be transmitted by PI3K. MM cells were electroporated with stSGK3 or stEGFP siRNAs and kept in cell culture for 2 days in order to recover and to downregulate SGK3. Cells were then washed 3 times with PBS and resuspended in full medium without FBS. The concentration of FBS was then adjusted for the different test conditions and cell death determined after 3 additional days in culture (Fig 6). In contrast to effects described for the hepatocellular carcinoma cell line QGY-7701 [35] SGK3 knockdown had no influence on the serum dependence of any of the MM cell lines tested (Fig 6).

### Relevance of SGK3 for the growth and survival of MM cells

The PI3K/Akt system is generally recognised as an important signalling and survival axis in tumor biology, including in MM [10,15,43,44,45], but clinically approved targeted molecular therapies are currently still limited to inhibitors of the mTOR components [17]. However, a large number of candidate compounds including isoform-specific PI3K inhibitors as well as pan-PI3K or Akt inhibitors, and drugs exhibiting dual target specificities towards PI3K/mTOR are in preclinical and clinical evaluation [46]. A phase III trial in MM with perifosine, an alkylphospholipid that leads to downregulation of Akt [47], has failed to show additional therapeutic benefit when the drug was combined with bortezomib and dexamethasone. However, a recent phase I trial with the pan-Akt inhibitor GSK2110183 (afuresertib) revealed clinically relevant responses in some MM patients [18], thus suggesting that Akt blockade can be beneficial if the right patients and therapeutic settings can be identified. Because our preclinical analyses of the Akt and PI3K isoforms in MM have revealed strong variability in the intrinsic activity of this pathway and in the response of MM cells to its blockade [10,15], we tested if SGK3 might provide a significant escape route from PI3K or Akt blockade-mediated apoptosis. SGK3 has recently been described as an oncogenic player in some solid cancers. Most prominently, it was identified as a crucial component in PI3K downstream signalling in cancer cells that harbour activating *PIK3CA* mutations, a condition that is often associated with relatively low intrinsic Akt activity [22]. SGK3 has also been described as androgen or estrogen inducible survival kinase in prostate [36] and breast cancer [34], respectively, and the *SGK3* locus constitutes a genetically amplified lesion in hepatic cancer [35]. However, these examples also show that for SGK3 no consistent oncogenic mechanism is implied, and an immunohistochemical study in ovarian cancer did not find SGK3 phosphorylation (activation) to be correlated to either the phospho-Akt status or the *PIK3CA* mutation status [48].

In our experiments we detected SGK3 protein expression in all MM cell lines and primary MM samples tested, but our functional analyses found no evidence for a substantial role of this kinase in the maintenance of growth and survival of MM cells. A clear limitation of our study is the lack of functional analyses with primary MM cells, which have been impossible due to the absence of suitable pharmacologic inhibitors, the non-transfectability of primary MM cells and a general dearth in unambiguous functional readouts for intrinsic or extrinsic SGK3 activation. However, the four transfectable MM cell lines chosen for our experiments do represent a broad range of intrinsic PI3K/Akt activation levels, and they represent MM cell lines that strongly (MM.1s), moderately (L-363, JJN-3) or not at all respond with apoptosis/viability decreases to blockade of Akt or PI3K/PI3K-p110α [10,11,15]. L-363 also represents the rare case of an MM cell line with an activating *PIK3CA* mutation. Furthermore, stealth siRNA-mediated transient SGK3 knockdown led to reliable and strong SGK3 protein depletion for more than 5 days, and thus permitted the addition of small molecule pathway inhibitors to MM samples virtually devoid of SGK3. Based on previous experience with Akt and PI3K-p110α blockade the time frame for these experiments should have been sufficient to establish functional effects [10,11,15]. The fact that SGK3 depletion alone or in combination with Akt inhibition failed to show an SGK3-dependent contribution to the survival or viability of MM cells therefore argues against a prominent role for SGK3 in the oncogenic deregulation of the PI3K/Akt system in multiple myeloma, and against its utility as a potential therapeutic target in this disease. Based on the limited scope of our study it can certainly not be ruled out that either in a clinical setting or perhaps for smaller MM subgroups SGK3 blockade might still prove beneficial. However, at the current stage of SGK inhibitor development no options exist to rigorously assess this.

## References

[1] Kuehl WM, Bergsagel PL. Molecular pathogenesis of multiple myeloma and its premalignant precursor. J Clin Invest. 2012;122:3456–3463. 10.1172/JCI61188 23023717 PMC3461901

[2] Moreau P, San Miguel J, Ludwig H, Schouten H, Mohty M, Dimopoulos M, et al Multiple Myeloma: ESMO Clinical Practice Guidelines for diagnosis, treatment and follow-up. Ann Oncol. 2013;24:Suppl.6:vi133–vi137. 10.1093/annonc/mdt297 23956208

[3] Kumar SK, Dispenzieri A, Lacy MQ, Gertz MA, Buadi FK, Pandey S, et al Continued improvement in survival in multiple myeloma: changes in early mortality and outcomes in older patients. Leukemia. 2014;28:1122–1128. 10.1038/leu.2013.313 24157580 PMC4000285

[4] Usmani SZ, Crowley J, Hoering A, Mitchell A, Waheed S, Nair B, et al Improvement in long-term outcomes with successive Total Therapy trials for multiple myeloma: are patients now being cured? Leukemia. 2013;27:226–232. 10.1038/leu.2012.160 22705990 PMC3744094

[5] Morgan GJ, Walker BA, Davies FE. The genetic architecture of multiple myeloma. Nat Rev Cancer. 2012;12:335–348. 10.1038/nrc3257 22495321

[6] Lohr JG, Stojanov P, Carter SL, Cruz-Gordillo P, Lawrence MS, Auclair D, et al Widespread genetic heterogeneity in multiple myeloma: implications for targeted therapy. Cancer Cell. 2014;25:91–101. 10.1016/j.ccr.2013.12.015 24434212 PMC4241387

[7] Leich E, Weißbach S, Klein HU, Grieb T, Pischimarov J, Stühmer T, et al Multiple myeloma is affected by multiple and heterogeneous somatic mutations in adhesion- and receptor tyrosine kinase signaling molecules. Blood Cancer J. 2013;3:e102 10.1038/bcj.2012.47 23396385 PMC3584721

[8] Hsu J, Shi Y, Krajewski S, Renner S, Fisher M, Reed JC, et al The AKT kinase is activated in multiple myeloma tumor cells. Blood. 2001;98:2853–2855. 11675360 10.1182/blood.v98.9.2853

[9] Pene F, Claessens YE, Muller O, Viguie F, Mayeux P, Dreyfus F, et al Role of the phosphatidylinositol 3-kinase/Akt and mTOR/P70S6-kinase pathways in the proliferation and apoptosis in multiple myeloma. Oncogene. 2002;21:6587–6597. 12242656 10.1038/sj.onc.1205923

[10] Zöllinger A, Stühmer T, Chatterjee M, Gattenlöhner S, Haralambieva E, Müller-Hermelink H-K, et al Combined functional and molecular analysis of tumor cell signaling defines 2 distinct myeloma subgroups: Akt-dependent and Akt-independent multiple myeloma. Blood. 2008;112:3403–3411. 10.1182/blood-2007-11-119362 18635812

[11] Steinbrunn T, Stühmer T, Sayehli C, Chatterjee M, Einsele H, Bargou RC. Combined targeting of MEK/MAPK and PI3K/Akt signalling in multiple myeloma. Br J Haematol. 2012;159:430–440. 10.1111/bjh.12039 22985491

[12] Munugalavadla V, Mariathasan S, Slaga D, Du C, Berry L, Del Rosario D. The PI3K inhibitor GDC-0941 combines with existing clinical regimens for superior activity in multiple myeloma. Oncogene. 2014;33:316–325. 10.1038/onc.2012.594 23318440

[13] Ramakrishnan V, Kimlinger T, Haug J, Painuly U, Wellik L, Halling T. Anti-myeloma activity of Akt inhibition is linked to the activation status of PI3K/Akt and MEK/ERK pathway. PLoS One. 2012;7:e50005 10.1371/journal.pone.0050005 23185517 PMC3503708

[14] Tu Y, Gardner A, Lichtenstein A. The phosphatidylinositol 3-kinase/AKT kinase pathway in multiple myeloma plasma cells: roles in cytokine-dependent survival and proliferative responses. Cancer Res. 2000;60:6763–6770. 11118064

[15] Hofmann C, Stühmer T, Schmiedl N, Wetzker R, Mottok A, Rosenwald A, et al PI3K-dependent multiple myeloma cell survival is mediated by the PIK3CA isoform. Brit J Haematol. 2014;166:529–539. 10.1111/bjh.12920 24766330

[16] Ismail SI, Mahmoud IS, Msallam MM, Sughayer MA. Hotspot mutations of PIK3CA and AKT1 genes are absent in multiple myeloma. Leuk Res. 2009;34:824–826. 10.1016/j.leukres.2009.11.018 20022634

[17] Porta C, Paglino C, Mosca A. Targeting PI3K/Akt/mTOR signaling in cancer. Front Oncol. 2014;4:64 10.3389/fonc.2014.00064 24782981 PMC3995050

[18] Spencer A, Yoon S-S, Harrison SJ, Morris SR, Smith DA, Brigandi RA, et al The novel AKT inhibitor afuresertib shows favorable safety, pharmacokinetics, and clinical activity in multiple myeloma. Blood. 2014;124:2190–2195. 10.1182/blood-2014-03-559963 25075128 PMC4229853

[19] Bruhn MA, Pearson RB, Hannan RD, Sheppard KE. Akt-independent PI3-K signaling in cancer—emerging role for SGK3. Cancer Manag Res. 2013;5:281–292. 10.2147/CMAR.S35178 24009430 PMC3762672

[20] Lang F, Böhmer C, Palmada M, Seebohm G, Strutz-Seebohm N, Vallon V. (Patho)physiological significance of the serum- and glucocorticoid-inducible kinase isoforms. Physiol Rev. 2006;86:1151–1178. 17015487 10.1152/physrev.00050.2005

[21] Fagerli U-M, Ullrich K, Stühmer T, Holien T, Köchert K, Holt RU, et al Serum/glucocorticoid-regulated kinase 1 (*SGK1*) is a prominent target gene of the transcriptional response to cytokines in multiple myeloma and supports the growth of myeloma cells. Oncogene. 2011;30:3198–3206. 10.1038/onc.2011.79 21478911

[22] Vasudevan KM, Barbie DA, Davies MA, Rabinovsky R, McNear CJ, Kim JJ, et al AKT-independent signaling downstream of oncogenic *PIK3CA* mutations in human cancer. Cancer Cell. 2009;16:21–32. 10.1016/j.ccr.2009.04.012 19573809 PMC2752826

[23] Manning BD, Cantley LC. AKT/PKB signaling: Navigating downstream. Cell. 2007;129:1261–1274. 17604717 10.1016/j.cell.2007.06.009 PMC2756685

[24] Fruman DA, Rommel C. PI3K and cancer: lessons, challenges and opportunities. Nat Rev Drug Discov. 2014;13:140–156. 10.1038/nrd4204 24481312 PMC3994981

[25] Burger R, Guenther A, Bakker F, Schmalzing M, Bernand S, Baum W, et al Gp130 and ras mediated signaling in human plasma cell line INA-6: a cytokine-regulated tumor model for plasmacytoma. Hematol J. 2001;2:42–53. 11920233 10.1038/sj.thj.6200075

[26] Uphoff CC, Drexler HG. Detecting Mycoplasma contamination in cell cultures by polymerase chain reaction. Methods Mol Med. 2004;88:319–326. 14634244 10.1385/1-59259-406-9:319

[27] Stühmer T, Arts J, Chatterjee M, Borawski J, Wolff A, King P, et al Preclinical anti-myeloma activity of the novel HDAC-inhibitor JNJ-26481585. Br J Haematol. 2010;149:529–536. 10.1111/j.1365-2141.2010.08126.x 20331455

[28] Logue SE, Elgendy M, Martin SJ. Expression, purification and use of recombinant annexin V for the detection of apoptotic cells. Nat Protoc. 2009;4:1383–1395. 10.1038/nprot.2009.143 19730422

[29] Steinbrunn T, Chatterjee M, Bargou RC, Stühmer T. Efficient transient transfection of human multiple myeloma cells by electroporation—an appraisal. PLoS One 2014;9:e97443 10.1371/journal.pone.0097443 24901949 PMC4047019

[30] Haan C, Behrmann I. A cost effective non-commercial ECL-solution for Western blot detections yielding strong signals and low background. J Immunol Methods. 2007;318:11–19. 17141265 10.1016/j.jim.2006.07.027

[31] Azab F, Vali S, Abraham J, Potter N, Muz B, de la Puente P, et al PI3KCA plays a major role in multiple myeloma and its inhibition with BYL719 decreases proliferation, synergizes with other therapies and overcomes stroma-induced resistance. Brit J Haematol. 2014;165:89–101. 10.1111/bjh.12734 24405121

[32] Wang Y, Xu W, Zhou D, Neckers L, Chen S. Coordinated regulation of serum- and glucocorticoid-inducible kinase 3 by a C-terminal hydrophobic motif and Hsp90-Cdc37 chaperone complex. J Biol Chem. 2014;289:4815–4826. 10.1074/jbc.M113.518480 24379398 PMC3931044

[33] Tessier M, Woodgett JR. Role of the Phox homology domain and phosphorylation in activation of serum and glucocorticoid-regulated kinase-3. J Biol Chem. 2006;281:23978–23989. 16790420 10.1074/jbc.M604333200

[34] Wang Y, Zhou D, Phung S, Masri S, Smith D, Chen S. SGK3 is an estrogen-inducible kinase promoting estrogen-mediated survival of breast cancer cells. Mol Endocrinol. 2011;25:72–82. 10.1210/me.2010-0294 21084382 PMC3089033

[35] Liu M, Chen L, Chan THM, Wang J, Li Y, Li Y, et al Serum and glucocorticoid kinase 3 at 8q13.1 promotes cell proliferation and survival in hepatocellular carcinoma. Hepatology. 2012;55:1754–1765. 10.1002/hep.25584 22262416

[36] Wang Y, Zhou D, Chen S. SGK3 is an androgen-inducible kinase promoting prostate cancer cell proliferation through activation of p70 S6 kinase and up-regulation of cyclin D1. Mol Endocrinol. 2014;28:935–948. 10.1210/me.2013-1339 24739041 PMC4042072

[37] Yan L. MK-2206: a potent oral allosteric AKT inhibitor. Proceedings of the 100th Annual Meeting of the American Association for Cancer Research (April 18–22, 2009, Denver, CO). 2009;Abstract Number: DDT01-1.

[38] Rehan M, Beg MA, Parveen S, Damanhouri GA, Zaher GF. Computational insights into the inhibitory mechanism of human AKT1 by an orally active inhibitor, MK-2206. PLoS One 2014;9:e109705 10.1371/journal.pone.0109705 25329478 PMC4201482

[39] Logie L, Ruiz-Alcaraz AJ, Keane M, Woods YL, Bain J, Marquez R, et al Characterization of a protein kinase B inhibitor in vitro and in insulin-treated liver cells. Diabetes. 2007;56:2218–2227. 17563061 10.2337/db07-0343

[40] Calleja V, Laguerre M, Parker PJ, Larijani B. Role of a novel PH-kinase domain interface in PKB/Akt regulation: structural mechanism for allosteric inhibition. PLoS Biol. 2009;7:e17 10.1371/journal.pbio.1000017 19166270 PMC2628406

[41] Casamayor A, Morrice NA, Alessi DR. Phosphorylation of Ser-241 is essential for the activity of 3-phosphoinositide-dependent protein kinase-1: identification of five sites of phosphorylation in vivo. Biochem J. 2001;342:287–292. PMC1220463 10455013

[42] Roberts DJ, Miyamoto S. Hexokinase II integrates energy metabolism and cellular protection: Akting on mitochondria and TORCing to autophagy. Cell Death Diff. 2015;22: 248–257. 10.1038/cdd.2014.173 PMC4291497 25323588

[43] Sahin I, Moschetta M, Mishima Y, Glavey SV, Tsang B, Azab F, et al Distinct roles of class I PI3K isoforms in multiple myeloma cell survival and dissemination. Blood Cancer J. 2014;4:e204 10.1038/bcj.2014.24 24769645 PMC4003418

[44] Keane NA, Glavey SV, Krawczyk J, O'Dwyer M. AKT as a therapeutic target in multiple myeloma. Expert Opin Ther Targets. 2014;18:897–915. 10.1517/14728222.2014.924507 24905897

[45] Mimura N, Hideshima T, Shimomura T, Suzuki R, Ohguchi H, Rizq O, et al Selective and potent Akt inhibition triggers anti-myeloma activities and enhances fatal endoplasmic reticulum stress induced by proteasome inhibition. Cancer Res. 2014;74:4458–4469. 10.1158/0008-5472.CAN-13-3652 24934808 PMC4140659

[46] Dienstmann R, Rodon J, Serra V, Tabernero J. Picking the point of inhibition: A comparative review of PI3K/AKT/mTOR pathway inhibitors. Mol Cancer Ther. 2014;13:1021–1031. 10.1158/1535-7163.MCT-13-0639 24748656

[47] Fensterle J, Aicher B, Seipelt I, Teifel M, Engel J. Current view on the mechanism of action of perifosine in cancer. Anticancer Agents Med Chem. 2014;14:629–635. 24628236 10.2174/1871520614666140309225912

[48] De Marco C, Rinaldo N, Bruni P, Malzoni C, Zullo F, Fabiani F, et al Multiple genetic alterations within the PI3K pathway are responsible for AKT activation in patients with ovarian carcinoma. PLoS One. 2013;8:e55362 10.1371/journal.pone.0055362 23408974 PMC3567053

